# Retraction: The immune response to lumpy skin disease virus in cattle is influenced by inoculation route

**DOI:** 10.3389/fimmu.2026.1814372

**Published:** 2026-02-27

**Authors:** Frontiers Editorial Office

The journal retracts the article cited above.

Following publication, concerns were raised regarding the conclusions of the article. An investigation was conducted in accordance with Frontiers policies. Retrospective analysis of blood samples revealed previously unknown pestivirus contamination in donor cattle directly inoculated with LSDV, and its impact on the immune response remains unknown. No pestivirus contamination was found in any of the ten arthropod-inoculated recipient cattle. Given extant concerns over the conclusions of the study, the article has been retracted. The authors wish to highlight to the research community the potential of pestivirus contamination of LSDV stocks.

This retraction was approved by the Chief Editors of Frontiers in Immunology and the Chief Executive Editor of Frontiers. The authors received a communication regarding the retraction and had a chance to respond. This communication has been recorded by the publisher.

